# Impact of Fat Distribution and Metabolic Diseases on Cerebral Microcirculation: A Multimodal Study on Type 2 Diabetic and Obese Patients

**DOI:** 10.3390/jcm13102900

**Published:** 2024-05-14

**Authors:** Regina Esze, László Balkay, Sándor Barna, Lilla Szatmáriné Egeresi, Miklós Emri, Dénes Páll, György Paragh, Liliána Rajnai, Sándor Somodi, Zita Képes, Ildikó Garai, Miklós Káplár

**Affiliations:** 1 Division of Metabolic Diseases, Department of Internal Medicine, Faculty of Medicine, University of Debrecen, Nagyerdei St. 98, H-4032 Debrecen, Hungary; pall.denes@unideb.hu (D.P.); paragh.gyorgy@med.unideb.hu (G.P.); dr.rajnai.liliana@unideb.hu (L.R.); somodi@med.unideb.hu (S.S.); mkaplar@belklinika.com (M.K.); 2 Kálmán Laki Doctoral School, Faculty of Medicine, University of Debrecen, Nagyerdei St. 98, H-4032 Debrecen, Hungary; 3 Division of Nuclear Medicine and Translational Imaging, Department of Medical Imaging, Faculty of Medicine, University of Debrecen, Nagyerdei St. 98, H-4032 Debrecen, Hungary; balkay.laszlo@med.unideb.hu (L.B.); barna.sandor@med.unideb.hu (S.B.); emri.miklos@med.unideb.hu (M.E.); kepes.zita@med.unideb.hu (Z.K.); garai@internal.med.unideb.hu (I.G.); 4 Division of Radiology and Imaging Science, Department of Medical Imaging, Faculty of Medicine, University of Debrecen, Nagyerdei St. 98, H-4032 Debrecen, Hungary; egeresi.lilla@med.unideb.hu; 5 Department of Medical Clinical Pharmacology, Faculty of Medicine, University of Debrecen, Nagyerdei St. 98, H-4032 Debrecen, Hungary; 6 ScanoMed Ltd., Nuclear Medicine Centers, Debrecen, Nagyerdei St. 98, H-4032 Debrecen, Hungary

**Keywords:** brain, breath-holding index (BHI), carotid intima-media thickness (cIMT), C-peptide, fat tissue, microcirculation, single-photon emission computed tomography (SPECT), type 2 diabetes mellitus (T2DM), obesity

## Abstract

**Background**: Since metabolic diseases and atherosclerotic vascular events are firmly associated, herein we investigate changes in central microcirculation and atherosclerosis-related body fat distribution in patients with type 2 diabetes mellitus and obesity. **Methods:** Resting brain perfusion single-photon emission computed tomography (SPECT) imaging with Technetium-99m hexamethylpropylene amine oxime ([^99m^Tc]Tc-HMPAO SPECT) was performed, and the breath-holding index (BHI) and carotid intima-media thickness (cIMT) were measured to characterise central microcirculation. Besides CT-based abdominal fat tissue segmentation, C-peptide level, glycaemic and anthropometric parameters were registered to search for correlations with cerebral blood flow and vasoreactivity. **Results**: Although no significant difference was found between the resting cerebral perfusion of the two patient cohorts, a greater blood flow increase was experienced in the obese after the breath-holding test than in the diabetics (*p* < 0.05). A significant positive correlation was encountered between resting and provocation-triggered brain perfusion and C-peptide levels (*p* < 0.005). BMI and cIMT were negatively correlated (rho = −0.27 and −0.23 for maximum and mean cIMT, respectively), while BMI and BHI showed a positive association (rho = 0.31 and rho = 0.29 for maximum and mean BHI, respectively), which could be explained by BMI-dependent changes in fat tissue distribution. cIMT demonstrated a disproportional relationship with increasing age, and higher cIMT values were observed for the men. **Conclusions:** Overall, C-peptide levels and circulatory parameters seem to be strong applicants to predict brain microvascular alterations and related cognitive decline in such patient populations.

## 1. Introduction 

Cerebral blood flow alterations (CBFs) induced by atherothrombotic cerebrovascular diseases (CVDs) are being hailed as the major risk factors for the development of cognitive deterioration and dementia [1]. Given that CVDs and related mortality rates impose an immense burden on societies worldwide, exhaustive research has been conducted into the investigation of such diseases in various patient populations [2,3,4].

Even though the available literature findings seem controversial, a wide array of clinical evidence confirms the association between cerebrovascular impairments and metabolic diseases including type 2 diabetes mellitus (T2DM) and obesity [5,6,7,8,9]. Hyperglycaemia and the appearance of ischaemia-related pathological cerebrovascular alterations were reported to be related in the context of T2DM [5,10]. Furthermore, obesity is also firmly connected with brain perfusion abnormalities [11]. 

Although the molecular background of the development of microcircular cerebral alterations induced by metabolic disorders is poorly understood, various pathophysiological factors associated with T2DM and obesity might explain the connection between central microvascular impairments and both T2DM and obesity. Impaired endothelium-dependent vasodilation and fibrinolytic dysfunction—generated by hyperglycaemia, insulin resistance, abnormal insulin signalling, chronic inflammation and obesity-derived metabolic derangements—project the foreseeable emergence of cerebrovascular abnormalities [12,13].

According to epidemiological data, several traditional risk factors could be responsible for the development of CVDs in metabolic diseases; examples include but are not limited to hypertension, dyslipidaemia, smoking, aging or weight gain [14,15,16,17]. Considering the ever-increasing prevalence of both T2DM and obesity, a rising number of studies has been spawned to investigate “non-traditional” vascular dangers in relation to metabolic disorders, including haemorheological laboratory parameters or the distribution of visceral and subcutaneous adipose fat tissue. Based on recent findings, the manifestation of microvascular complications in type 1 diabetic patients might be attributable to impaired C-peptide secretion [18,19]. Additionally, the presence of increased amounts of visceral type of adipose tissue has been associated with metabolic and atherosclerotic alterations [20]. 

Since T2DM and obesity-associated subclinical brain microvascular changes may indicate the development of neurological disturbances, the timely diagnostic assessment of cerebrovascular impairments is of paramount importance [21]. Although in current clinical practice, computed tomography (CT) and magnetic resonance imaging (MRI) provide useful means for the examination of cerebral structural abnormalities, single-photon emission-computed tomography (SPECT) has recently emerged as a technique for evaluating brain perfusion and functional deteriorations [22]. Nevertheless, its widespread clinical implementation has been hampered by the related financial burden and radiation exposure. Képes et al. confirmed the feasibility of Technetium-99m hexamethylpropylene amine oxime ([^99m^Tc]Tc-HMPAO) SPECT in the assessment of the cerebral perfusion of T2DM and obese individuals [23]. Considering that carotid ultrasonography (CUS) correctly represents the severity of the atherosclerotic lesions, including intima-media thickness (IMT) and the presence of atherosclerotic plaques, stenosis or occlusion in the carotid arteries, its addition to the diagnostic workup of T2DM and obesity has clear advantages. As the common carotid artery IMT measured by B-mode CUS correlated well with pathological findings, IMT is regarded as a quantitative and reproducible marker of carotid atherosclerosis [24,25,26]. Furthermore, to quantify provocation-triggered regional CBFs as well as the functional reserve capacity of the cerebral vasculature, transcranial Doppler (TCD)-mediated breath-holding test (BHT) is recommended [27,28].

As T2DM and obesity-induced microvascular alterations could potentially lead to cognitive decline or even manifest dementia, the in-depth assessment of cerebral microcirculation in metabolic disturbances is becoming a central issue in current medical research. Discoveries derived from such investigations would not only contribute to broaden the horizon of the existing knowledge on the association between metabolic diseases and microcircular alterations, but also open a new era towards the development of new targets for therapeutic interventions and drug design. 

Applying [^99m^Tc]Tc-HMPAO SPECT, CUS and TCD examinations, herein, we aimed at assessing and comparing the central microcirculation of patients with T2DM and obesity, and searching for correlations between cerebral perfusion and anthropometric and CT-based abdominal fat segmentation data, as well as different laboratory parameters characterising glucose homeostasis.

## 2. Materials and Methods

### 2.1. Study Participants

Our multimodal study was accomplished with the involvement of 52 (32 men and 20 women) T2DM and 47 (21 men and 26 women) obese patients recruited from the Department of Internal Medicine, Faculty of Medicine, University of Debrecen (Debrecen, Hungary) and from a private general medical praxis of the city of Miskolc (Miskolc, Borsod-Abaúj-Zemplén county, Hungary). The following strict inclusion criteria were applied to patient selection: aged between 18 and 70 and an absence of anamnestic data indicating mental or brain disorders. Included participants were categorised into two groups: those diagnosed with obesity (BMI > 30 kg/m^2^) without T2DM and those with anamnesis indicating regularly controlled T2DM, fulfilling the actual diagnostic guidelines for Hungary at the time of examination (Clinical Professional Guideline—On the diagnosis of diabetes mellitus, the antihyperglycaemic treatment and the care of the diabetics in adulthood. In: Diabetologia Hungarica, Ministry of Human Resources—State Secretariat for Health, Health Professional College, Hungary, 2017; Volume 25, Issue 1, pp. 1–75.), regardless of their BMI. Exclusion criteria were as follows: gravidity, breastfeeding, presence of acute or chronic inflammatory and liver disease, oral steroid intake or retinoid treatment, diagnosed hyperthyroidism or the presence of uncontrolled hypothyroidism, history of malignant diseases except for basocellular carcinoma, existence of crural ulcer at the time of the investigation, long-term anticoagulant treatment or change in regular medical therapy six months before the start of the study. 

Prior to enrolment, the participants were provided with detailed pieces of information regarding the major aims of the research and the performed examinations. Informed consent was collected from all the study patients engaged in the research (OGYEI/2829-4/2017).

### 2.2. Assessment of Anthropometric Parameters

The following anthropometric parameters were determined: height (centimetre/cm), body weight (BW; kgs), body mass index (BMI; kg/m^2^) and age (years). Body height—expressed in cm—was measured in a standing position with shoulders in normal alignment without shoes on the outpatient medical scales. We defined BW in light clothing without shoes applying a standard digital scale and values were recorded to the nearest 100 g. BMI was calculated as weight in kilograms (kg) divided by height in metres squared (m^2^). The age of all the participants was registered as well. 

### 2.3. Measurement of Laboratory Parameters

Following an 8 h long fasting period, all patients were subjected to comprehensive laboratory examinations. As shown in Table 1, the following four laboratory markers assessing the glucose homeostasis of the patients were measured: plasma glucose, glycated haemoglobin (HbA1c), insulin and C-peptide. Plasma samples containing sodium fluoride-potassium oxalate (NaF–KOx) were used for the determination of blood glucose levels (reference range: 3.6–6 mmol/L). High-performance liquid chromatography (BioRad, Hercules, CA, USA) was applied for the measurement of HbA1c (reference range: 4.2–6.1%) levels from K3-EDTA anticoagulated whole blood samples, while insulin (4.3–20 mU/L) and C-peptide (reference range: 350–1170 pmol/L) concentrations were determined from native plasma samples.

### 2.4. Measurements of Carotid Intima-Media Thickness (cIMT-IMT)

For intima-media thickness (IMT) determination, Philips HD 11 XE ultrasound equipment with a 7.5 MHz linear transducer was used. At the end of diastole, the image was magnified and frozen, and IMT was determined 10 mm proximal to the carotid bulb on the wall of the common carotid arteries farthest from the transducer on both sides with the transducer in the medio-lateral direction. IMT (expressed in mm) was measured per millimetre on a 1 cm long segment of both arteries; therefore, 10 measurements were recorded separately for the right and the left common carotid arteries. Out of the 20 IMT figures, we registered the mean and the maximum in case of all patients [29,30]. 

### 2.5. Estimation of Cerebrovascular Reactivity (CVR) based on Breath-Holding Test

Transcranial Doppler (DWL, Multi-Dop X, S. No: MDX-1156) examinations [31,32] with a 2 MHz probe fixed to the temporal window were accomplished to perform the breath-holding test (BHT) at rest. To determine the resting blood flow parameters, the probe was placed on the temporal window, and the mean flow velocity (MFV) was quantified at either of the following test depths in the middle carotid artery (MCA) of both sides: 45, 50 and 55 mm. The identification of the optimal acoustic window was confirmed by the registration of a pulse wave of adequate shape. Afterwards, patients were asked to hold their breath for 30 s to measure their MCA velocity profiles before (MFVbaseline) and at the end of (MFVend) a 30 s breath hold. The breath-holding index (BHI) was calculated using the following formula:

BHI = [(MFVend − MFVbaseline)/MFVbaseline] × 100/breath-holding time (s)

Physiologically, BHI-induced cerebral blood flow enhancement exceeds 30% in healthy adults. BHI values between 20 and 30% indicate decreased cerebrovascular reserve capacity, while a BHI value of less than 20% refers to the absence of cerebral vasomotor reactivity [32].

### 2.6. Brain Perfusion Assessment with Single-Photon Emission Computed Tomography (SPECT) at Rest

**Figure 1:**
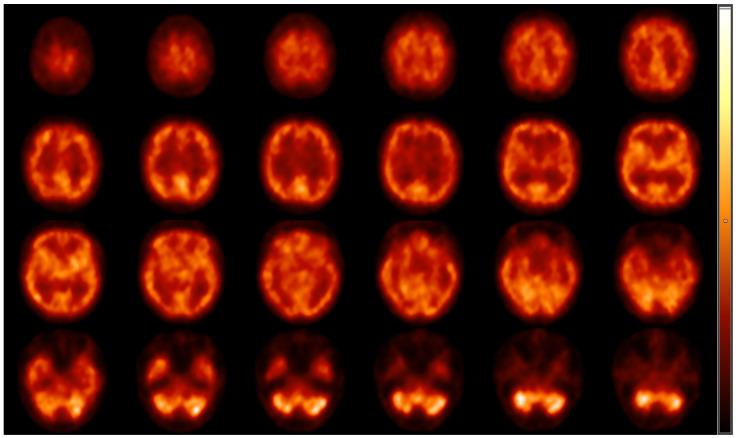
Representative transaxial [^99m^Tc]Tc-HMPAO brain perfusion SPECT images acquired at rest. Following the intravenous administration of 740 MBq of [^99m^Tc]Tc-HMPAO, dynamic SPECT acquisition was performed using AnyScan S Flex SPECT camera (Mediso Ltd., Budapest, Hungary). No perfusion abnormalities can be detected on the slices; the right and the left hemispheres show symmetrical and consistent radiotracer accumulation. High [^99m^Tc]Tc-HMPAO is shown in the cortical grey matter, the region of the basal ganglia and in the visual cortex, while the white matter and the ventricles are presented with faint radioactivity. HMPAO, hexamethylpropylene amine oxime; SPECT, single-photon emission computed tomography.

For the evaluation of resting cerebral brain perfusion, we applied a dual-head gamma camera equipped with low-energy, high-resolution parallel hole collimators (AnyScan S Flex, Mediso Ltd., Budapest, Hungary). To prohibit the radiotracer uptake of the thyroid glands, a perchlorate capsule was orally administered to all subjects 30 min prior to radiopharmaceutical injection. After a 10 min rest in a dimly lit room, approximately 740 MBq of [^99m^Tc]Tc-HMPAO was intravenously injected into the right cubital vein of the study participants lying under the camera. Afterwards, SPECT acquisition was conducted with the following imaging parameters: 120 views, 128 × 128 matrix size, 2.36 mm pixel size, 30 s projection time and auto body contour. Representative transaxial brain perfusion SPECT images acquired at rest can be seen in Figure 1. Among the study participants, 6 did not participate in the perfusion SPECT imaging process.

### 2.7. Abdominal Fat Tissue Segmentation 

To quantify the ratio of visceral (VAT) and subcutaneous (SAT) abdominal fat tissue, transaxial low-dose CT images were performed using the CT compartment of the AnyScan positron emission tomography/computed tomography (PET/CT; Mediso Ltd., Budapest, Hungary) hybrid device with the following CT parameters: 120 kW and 100 mAs. 

**Figure 2:**
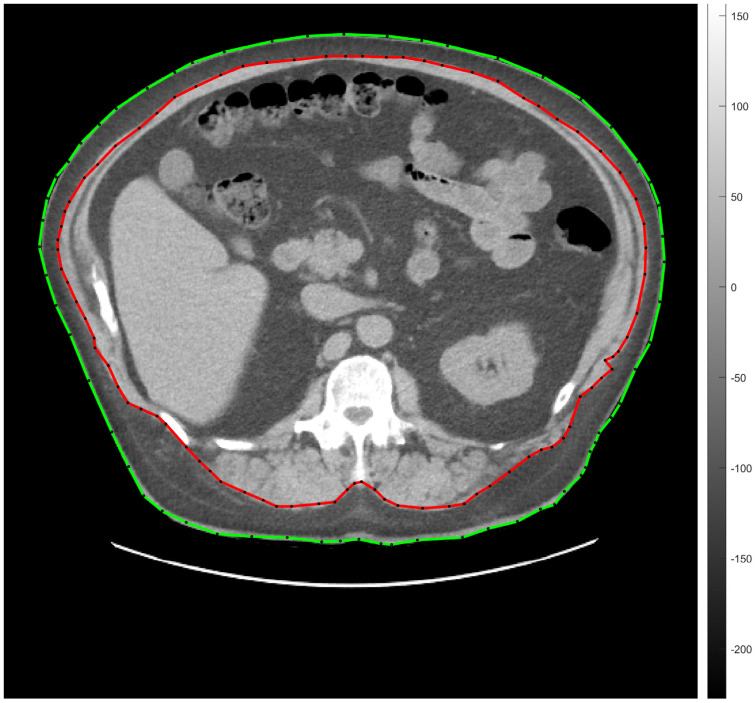
Transaxial low-dose CT slice acquired at the level of vertebra L1 for the representation of semi-automatic abdominal fat tissue segmentation. The green line indicates the body contour, the red one is for the abdominal cavity. SAT and VAT were calculated by counting the segmented pixels between the green and the red ROIs, and within the area of the red ROI, respectively. CT, computed tomography; L1, vertebra lumbar I; SAT, subcutaneous adipose tissue; ROI, region of interest; VAT, visceral adipose tissue.

Abdominal fat segmentation was carried out by a semi-automatic method. Briefly, a transaxial CT slice—acquired at the level of vertebra L1—was chosen for image processing [33]. Thereafter, ROIs (regions of interest) representing the body contour (green line) and the abdominal cavity (red line) were manually deposited on the selected CT scans (seen on representative Figure 2).

Fat tissue segmentation was performed according to the −190 and −30 HU (Hounsfield unit) range of attenuation. The amount of SAT and VAT was determined on the basis of the number and the volume of the segmented pixels measured between the red and the green lines (SAT) and under the red line (VAT). Seven of the involved participants did not consent to low-dose CT imaging.

### 2.8. Statistical Analyses

Because many parameters have non-Gaussian distributions, we performed a non-parametric Wilcoxon rank sum test to examine group differences and Spearman correlation tests to investigate the monotonous association between parameter pairs. For statistical analysis, table and figure generation, we developed R (version 4.3.2) software.

## 3. Results

### 3.1. Group Comparison

#### 3.1.1. Anthropometric Parameters

The main anthropometric characteristics of the involved subjects showed no significant differences between the age (*p* = 0.47) and sex (*p* = 0.15) of the two groups. Mean age appeared to be 50.71 ± 7.74 and 51.53 ± 9.68 years in the T2DM and in the obese group, respectively. Considerably higher BMI values were registered in the obese group compared to the diabetic one, with the respective mean BMI figure being 38.07 ± 6.06 and 33.57 ± 5.86 in the former and in the latter groups (*p* < 0.001). 

#### 3.1.2. Glucose Homeostasis

Upon the assessment of the glucose homeostasis of the patients, we observed that the fasting plasma glucose concentrations notably differed between the two study groups (*p* < 0.001) with corresponding values being 8.86 ± 3.14 mmol/L, 5.45 ± 0.55 mmol/L for the T2DM and the obese cohort. Similarly, a meaningful difference was found between the HbA1c levels of the two groups, as follows: 7.56 ± 1.25% and 5.51 ± 0.32% in the diabetic and obese study groups. Besides more elevated C-peptide values (*p* < 0.05), the presence of hyperinsulinaemia was also more apparent among the obese participants than in the subjects with T2DM (*p* = 0.051). The HOMA-IR (Homeostatic Model Assessment for Insulin Resistance) index used for the characterisation of insulin resistance was above the normal range in both groups (5.87 ± 5.47 and 4.27 ± 2.72 for the diabetics and the obese, respectively) and no significant difference was found between the two groups (*p* = 0.519). These results are demonstrated in Table 1.

#### 3.1.3. Resting Cerebral Blood Perfusion (CBP), Breath-Holding Test (BHT) and Intima-Media Thickness (IMT)

**Figure 3:**
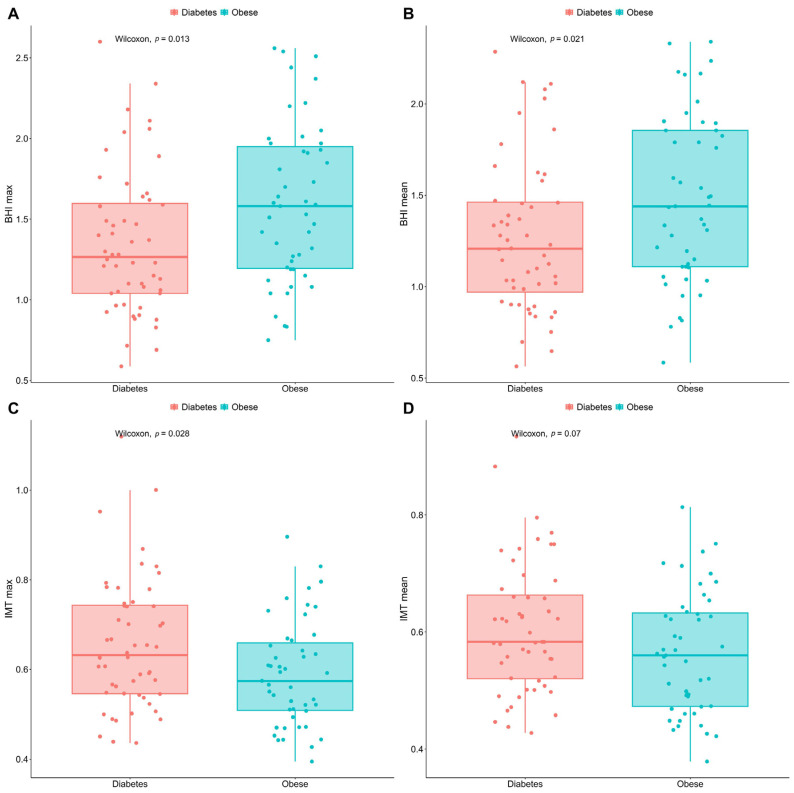
Box-and-whisker plots comparing the max. and mean BHI (**A**,**B**) and IMT (**C**,**D**) values of T2DM and non-DM obese patients. BHI figures were calculated after breath-holding test. Points in red are for the diabetics, in green for people with obesity. The significance was set at *p* < 0.05. Number of involved patients (**A**–**D**): n = 99. BHI, breath-holding index; DM, diabetes mellitus; max., maximum; T2DM, type 2 diabetes mellitus; IMT, intima-media thickness.

No significant disparity could be detected between the resting cerebral blood perfusion (CBP) of the diabetic and the obese patients (*p* > 0.05); however, after breath-holding test (BHT)-based provocation, a more pronounced blood flow increase was recorded in the obese subgroup relative to the T2DM group (*p* < 0.05, as seen in Figure 3A,B). The T2DM patients were characterised by remarkably higher maximum IMT values than the obese group (*p* < 0.05). Although the group difference regarding the mean intima-media thickness (IMT) values was close to the threshold (*p* = 0.07), no considerable difference could be identified in statistical terms between the assessed groups. These results are displayed in Figure 3C,D.

### 3.2. Correlation Analyses

**Figure 4:**
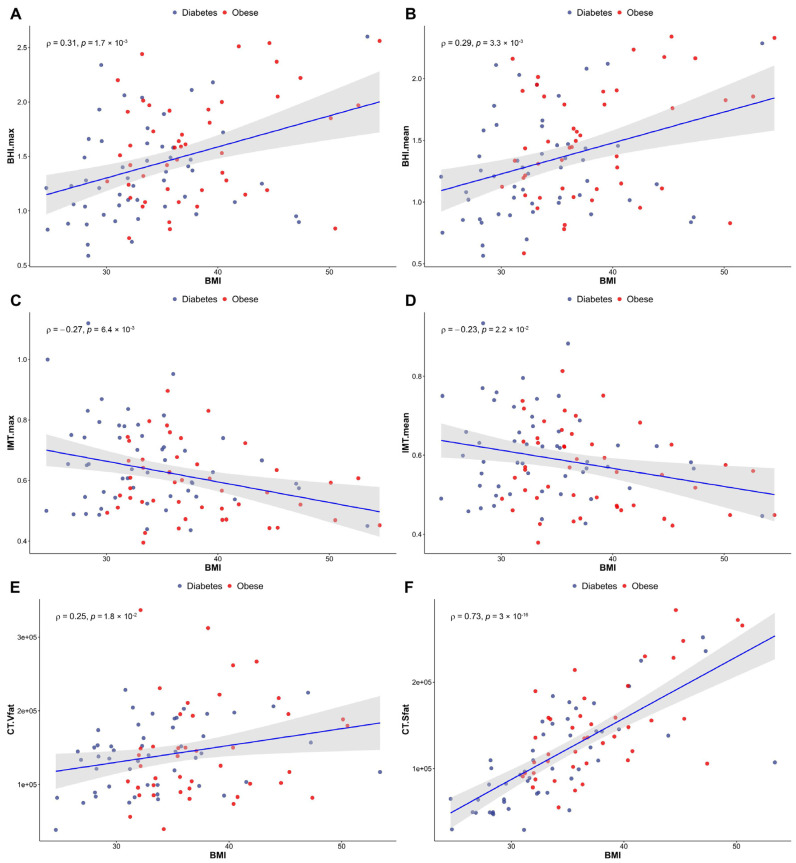
Correlations between BMI and both BHI (**A**,**B**) and IMT (**C**,**D**) in a study population including T2DM and obese patients. (**E**,**F**) demonstrate changes in abdominal fat distribution in relation to BMI. (**E**): visceral fat, (**F**): subcutaneous fat. Red spots indicate the obese patients, while blue ones are for the diabetics. Significance was set at *p* < 0.05. Number of involved patients (**A**–**D**): n = 99; (**E**,**F**): n = 92. BMI, body mass index; BHI, breath-holding index; IMT, intima-media thickness; T2DM, type 2 diabetes mellitus.

A considerable positive correlation was observed between BMI and the maximum (*rho* = 0.31)/mean (*rho* = 0.29) BHI values (*p* < 0.005, demonstrated in Figure 4A,B). Meanwhile, Figure 4C,D shows a notable negative correlation between BMI and the maximum (*p* < 0.01)/mean (*p* < 0.05) IMT figures (rho = −0.27 and −0.23 for maximum and mean IMT, respectively). In addition, the BHI values showed a decrease direct proportion to ageing (*p* < 0.005, rho = −0.29 and rho = −0.31 for maximum and mean BHI, respectively).

A significant positive correlation was found between the maximum (*rho* = 0.28)/mean (*rho* = 0.27) IMT and the amount of intra-abdominal visceral fat (*p* < 0.01), whereas the maximum (*rho* = −0.31)/mean (*rho* = −0.29) IMT values and the mass of subcutaneous fat tissue were negatively correlated with each other (*p* < 0.005 and *p* < 0.01 for maximum and mean IMT, respectively). An extremely strong positive correlation was detected between age and the maximum (*rho* = 0.46)/mean (*rho* = 0.46) IMT values (*p* < 0.001). 

Our results indicate a BMI-dependent increment of abdominal fat in both groups—that is, the higher the BMI, the greater the volume of the abdominal fat present. We noticed more significant elevation of the amount of metabolically more favourable subcutaneous fat compared to the visceral type of adipose tissue (rho = 0.25 and rho = 0.73 for visceral and subcutaneous fat, respectively), shown in Figure 4E,F. The numerical values of VAT and SAT obtained from low-dose CT imaging are included in Supplementary Material Table S1. 

Moreover, neither of the brain perfusion-related parameters showed an association with sex. On the contrary, however, a significant relationship was detected between sex and IMT, i.e., men had significantly higher IMT compared to women (*p* < 0.005). 

Although we encountered positive correlation between resting [^99m^Tc]Tc-HMPAO brain perfusion and BHT-based middle cerebral artery blood flow (*p* < 0.05, *r* = 0.26), no meaningful association was present between cerebral perfusion and IMT. The numerical values of the resting brain perfusion are detailed in Supplementary Material Table S1.

C-peptide levels and both resting (*p* < 0.005, *rho* = 0.31) and provocation-induced (*p* < 0.005, rho = 0.32 and 0.30 for maximum and mean BHI) cerebral microcirculation were reported to positively correlate. Contrarily, no association was found between IMT data and C-peptide levels (*p* > 0.05). Finally, there was no significant association identified between the insulin levels and the investigated parameters.

## 4. Discussion

Despite several advances that have been reported on the association between metabolic diseases and cerebral vascular alterations, there are still many facets that remain to be fully understood. Taking the rising incidence of T2DM/obesity and related microvascular complications into account, further investigation of the pathophysiology and the breadth of their manifestation in the central microcirculation is among the top priorities of current research. Using different approaches, we therefore intended to explore and compare the cerebral microvascular pattern of a selected group of T2DM and obese patients, as well as to seek associations with various biochemical and anthropometric parameters. 

### 4.1. Group Comparison

Upon SPECT image assessment, no significant disparity was found between the regional cerebral perfusion of the obese and the type 2 diabetic cohort. Although future studies are warranted to fully uncover the reason behind this, we assume that it could possibly be due to the fact that the diabetic patients were under strict and regular medical control that aimed to prevent the development of diabetes-induced brain perfusion changes. In contrast to our results, however, Képes et al. registered a considerable perfusion difference between T2DM and obese subjects in the region of the insula applying [^99m^Tc]Tc-HMPAO SPECT imaging [23]. Although only a few pieces of research related to the comparison of the brain perfusion of diabetic and obese individuals are available so far, there are some research findings on the CBF of patients with metabolic syndrome, obesity or diabetes. A 15% lower mean grey matter blood flow was registered by Birdsill and co-workers in patients with metabolic syndrome compared to controls [34]. Cui et al. encountered decreased CBF in the posterior cingulate cortex, precuneus and bilateral occipital lobe of T2DM patients [10]. Studying healthy subjects, the group of Willeumier associated diminished regional CBF with elevated BMI values in several Broadmann areas (8, 9, 10, 11, 32, 44) and in the prefrontal cortex [35]. Similarly, Képes et al. also reported high BMI-triggered hypoperfusion in the brain stem that could be in connection with obesity-related pathophysiological processes such as leptin resistance or impaired insulin signalling [23]. Further, according to the literature data, cerebral perfusion impairments induced by metabolic disorders may result in neurodegenerative changes in the long run. For this reason, timely diagnostic assessment of cerebral perfusion abnormalities related to either obesity or T2DM is of crucial importance to delay or even prevent the occurrence of disease-generated brain alterations. 

Breath-holding provocation-induced improved cerebral vasoreactivity as well as lower max. IMT values of the obese participants compared to the diabetics may lead to the conclusion that the development of diabetes-associated pathological microvascular changes has already begun in the enrolled T2DM group. Correspondingly, Tchistiakova and colleagues also strengthened worse cerebrovascular reactivity in diabetes than in individuals with hypertension [2]. Furthermore, reduced mean blood flow velocity/volume (BFV) and impaired carbon dioxide inhalation reactivity were pointed out in a T2DM patients compared to the healthy control in a study conducted by Novak and co-workers, that was also consistent with our observations [36]. Although unlike in the present study acetazolamide was applied for provocation to assess the cerebral vascular autoregulation of subjects with impaired glucose homeostasis, Selvarajah et al. determined notably lower vasoreactivity in the internal carotid artery of patients with T2DM and IGT than in the control cohort [37]. 

Considering that BHI is supposed to be the strongest independent predictor of cognitive impairment, its measurement should be added to the workup and treatment planning of diabetic patients [38]. 

### 4.2. Correlations between the Assessed Metabolic and Vascular Parameters

In the second part of our study, we performed correlation analyses between the investigated parameters. While BMI showed positive correlation with BHI, a significant negative association was registered between BMI and IMT. Unexpectedly, these results imply that an increase in BMI paradoxically had a positive effect on brain reactivity as well as on carotid IMT in this selected group of patients with metabolic abnormalities. We hypothesise that factors related to microvascular molecular mechanisms and angiogenic processes may underly our observations. Our finding is in contradiction with the currently available findings regarding the relationship between BMI and IMT. Liu et al. reported that BMI was in positive correlation with IMT in patients with cerebrovascular disorders [39]. In the study of Rodríguez-Flores et al., however, BMI was negatively associated with cerebrovascular reactivity [40]. Comparing the vasomotor reactivity of 85 non-obese (BMI ≤ 27 kg/m^2^) and 85 obese participants (BMI ≥ 35 kg/m^2^) without diabetes mellitus and hypertension, they found notably lower BHI in the obese cohort than in the control group. Although due to the lack of healthy control group in our study the results of Rodríguez-Flores and co-workers cannot really be compared with those of our study, we suppose that the contradictive findings may be attributable to the differences between the number and the characteristics of the study subjects, as well as the applied methods for BHI determination. In further contrast to our findings, a positive correlation (r = 0.170)—supported by univariate regression analysis—was registered between BMI and mean carotid IMT in research conducted by Sugiura et al. who explored the connection between obesity-linked parameters and subclinical atherosclerosis in 7750 employees with no anamnestic data of cardiovascular events or treatments [41].

In a bid to better understand the correlations related to BMI, the visceral and subcutaneous abdominal fat distribution pattern of the participants was determined using low-dose CT. We pointed out that a gradual increase in BMI anticipated the deposition of metabolically neutral subcutaneous adipose tissue. Contrarily, more pronounced accumulation of visceral type of fat tissue—associated with various metabolic and atherosclerotic disorders—characterised patients with lower BMI values, which could underpin the experienced poorer cerebral circulatory parameters [20].

In addition, in a former study of Liu et al., a positive association was encountered between IMT and both the vascular and subcutaneous types of adipose tissue in patients without cerebrovascular diseases, which was partly in accordance with our findings [39]. Given the firm relationship between BMI and central circulation, our results highlight the importance of the regular monitoring of BMI in patients with metabolic diseases to follow the possible microvascular changes and alterations in the central nervous system. 

According to our measurements, we can conclude that age increase may be a risk factor not only for the decline of cerebral vascular reactivity but also for the thickening of the intima-media of the carotid artery wall. In line with our results, a strong association was found between increasing age and IMT abnormalities in a critically obese adult cohort by Ko et al. [42]. Evaluating the Doppler ultrasound (UH) results of 120 healthy volunteers, Zavoreo and Demarin reported the decrease of BHI in correlation with aging, which was also in agreement with our findings [28].

Moreover, our IMT results indicate that being of male sex may also increase the likelihood of the development of subclinical atherosclerosis; however, regarding the other assessed cerebral circulatory parameters, no significant sex-dependent difference was depicted. This is consistent with the outcomes of Qu et al., who also demonstrated considerably higher atherosclerosis-related cIMT values for men relative to women; moreover, these were associated with obesity-related anthropometric parameters, including BMI [43]. Identically to our measurements, in the [^99m^Tc]Tc-HMPAO study of Catafau and co-workers, sex did not affect the regional CBF of young and elderly subjects either; however, in particular brain regions, including the left frontal lobe and posterior region of the left temporal lobe, they detected hypoperfusion in the group of the elderly [44].

Finally, the significant positive correlation observed between C-peptide levels and brain circulatory parameters under provocation as well as at rest suggests that C-peptide has a crucial role in the improvement of cerebral microvascular function [45,46]. Based on the previous literature data, the interplay between C-peptide and the vascular and rheological components of the microcirculation contribute to better microcircular activity in type 1 diabetes mellitus [45]. Several studies confirmed that the maintenance of haemorheologically appropriate C-peptide levels is connected with meaningful metabolic benefits including vasodilatory effects or augmented endothelial function, indicating its significance in dysmetabolic states [18,19,45,46,47,48].

Furthermore, while in our study C-peptide was correlated only with cerebral blood perfusion, in the paper of Kim et al., the basal C-peptide levels of type 2 diabetic patients were positively associated with IMT values [49]. Although the exact reason behind the controversial findings is not exactly known, we suppose that in the study of Kim and co-workers, patients with a more severe stage of diabetes and related more elevated C-peptide levels as well as longer disease duration were enrolled compared to our subjects.

## 5. Conclusions—Future Outlook

Improved circulatory parameters experienced in relation to elevated C-peptide levels and breath-holding provocation show the value of these parameters as independent predictors of cognitive decline in such patient populations. The associations between the BMI values and the deposition of fat tissue (visceral vs. subcutaneous) draw attention to the clinical importance of the regular monitoring of anthropometric parameters during patient follow-up. Moreover, the more profound interpretation of the correlation regarding BMI increase and augmented cerebral reactivity is, however, part of comprehensive future work. 

In addition, the findings obtained from the present study may possibly lay the groundwork for the recognition of new treatment targets, leading to the ultimate goal of the establishment of personalised patient management. Overall, future studies are recommended to further expand our knowledge on cerebral deterioration in association with T2DM/obesity, which could be exploited in disease-related cerebrovascular risk reduction as well as in combating the development of cognitive decline or dementia in metabolic disturbances.

## Figures and Tables

**Table 1:** Laboratory parameters assessing the glycemic status of patients with obesity and type 2 diabetes mellitus.

Parameters	Type 2 Diabetic Participants (n = 52)		Non-DM, Obese Participants (n = 47)		Reference Range	*p* Value
	Mean	SD	Mean	SD		
HbA1c (%)	7.56	1.25	5.51	0.32	4.2–6.1%	<0.001
glucose (mmol/L)	8.86	3.14	5.45	0.55	3.6–6 mmol/L	<0.001
C-peptide (pmol/L)	765.77	453.94	864.43	395.70	350–1170 pmol/L	<0.05
insulin (mU/L)	14.21	10.87	17.34	10.18	4.3–20 mU/L	0.051
HOMA-IR	5.87	5.47	4.27	2.72	<4.0	0.519

DM, diabetes mellitus; SD, standard deviation; HbA1c, glycated haemoglobin; HOMA-IR, Homeostatic Model Assessment for Insulin Resistance.

## Data Availability

The dataset used and/or analyzed during the current study is available from the corresponding author upon reasonable request.

## Supplementary Materials

The following supporting information can be downloaded at: https://www.mdpi.com/article/10.3390/jcm13102900/s1, Table S1. Numeric data obtained from the primary imaging techniques (brain perfusion [^99m^Tc]Tc-HMPAO brain perfusion SPECT and low-dose abdominal CT). The table contains quantitative data—quantified in pixels—on brain perfusion (separately for the right and the left hemispheres) and visceral and subcutaneous adipose tissue.
