# Supplementary material for: Impact of Fat Distribution and Metabolic Diseases on Cerebral Microcirculation: A Multimodal Study on Type 2 Diabetic and Obese Patients
Source: J Clin Med. 2024 May 14;13(10):2900. doi: 10.3390/jcm13102900 (PMC11122647; doi:10.3390/jcm13102900)
Supplement: Supplementary file 1 [file jcm-13-02900-s001.zip › jcm-2977947-supplementary.pdf]

Table S1: Numeric data obtained from the primary imaging techniques (brain perfusion [99mTc]Tc-HMPAO brain perfusion SPECT and low-dose abdominal CT). The table contains quantitative data - quantified in pixels - on brain perfusion (separately for the right and the left hemispheres) and visceral and subcutaneous adipose tissue.

Patient Number	Brain perfusion of the left hemisphere n=93	Brain perfusion of the right hemisphere n=93	Visceral adipose tissue n=92	Subcutaneous adipose tissue n=92
1.	N.A.	N.A.	146250	53643.91
2.	57	55	165595.2	109276.2
3.	52	49	195836	142978.9
4.	43	48	163034.6	72393.05
5.	48	49	189441.7	52170.49
6.	53	52	202840.7	99305.6
7.	44	44	190648.1	88438.53
8.	37	37	82187.23	30388.68
9.	43	46	156935.8	235880.6
10.	46	44	152334.4	71115.13
11.	71	67	136360.4	124477.7
12.	45	47	197624.1	195259
13.	48	46	181302.1	109576.6
14.	N.A.	N.A.	144809.9	49967.51
15.	43	42	39381.78	65250.06
16.	51	53	121268.6	100440.5
17.	55	54	86726.7	99901.64
18.	57	54	80227.44	183867.5
19.	63	68	119151.5	176757.9
20.	45	47	89387.44	49819.69
21.	49	51	151705	82525.78
22.	47	44	N.A.	N.A.
23.	58	55	228513.5	92944.62
24.	64	66	177418.5	157455.1
25.	38	47	224551.3	251842.1
26.	42	42	103344.4	224808.5
27.	41	42	133456.5	49452.53
28.	50	48	142406.7	143222.1
29.	49	49	97221.83	64539.58
30.	51	51	139726.9	139383.6
31.	44	43	75358.96	64282.09

32.	48	45	153240.4	48928.01
33.	71	73	95800.86	78329.64
34.	49	51	82931,09	89106.1
35.	46	48	204531.8	85751.19
36.	45	52	99368.58	154408.2
37.	40	47	138043.7	47607.18
38.	52	60	144585.8	138353.6
39.	50	52	75726.12	29945.22
40.	44	45	98118.28	175623
41.	66	57	151824.2	169695.9
42.	46	48	150236.3	81548.27
43.	45	54	196169.8	72221.39
44.	34	37	173825.3	49962.74
45.	47	51	206068.9	138172.4
46.	49	50	135168.3	61874.07
47.	41	46	83989.67	109624.3
48.	45	57	85050.23	145613.5
49.	66	66	125547.2	158922.4
50.	42	45	120902.2	96639.49
51.	66	60	116874	107139.9
52.	54	52	N.A.	N.A.
53.	41	44	N.A.	N.A.
54.	58	58	131827.9	120855.4
55.	53	54	83344.55	120149.8
56.	41	41	110358.6	214051.1
57.	79	78	312302.9	129541.7
58.	46	49	108508.5	157217.2
59.	62	63	117077.2	157565.3
60.	58	55	89802.28	119723.7
61.	49	51	145568.1	151232.9
62.	48	49	124949.8	87551.62
63.	39	43	151523.8	108565.7
64.	42	41	150088.5	160764.8
65.	39	39	101027	229801
66.	44	47	139979.6	94871.04
67.	49	53	104426.8	106052.8
68.	52	61	195631	247720.4
69.	45	46	99300.83	151757.4
70.	45	49	94580.17	134987.1
71.	40	48	85639.52	158819.3
72.	N.A.	N.A.	195478.4	74648.47
73.	54	55	138525.3	147241.8
74.	50	51	336807.4	189665.8

75.	50	55	N.A.	N.A.
76.	57	56	74009.52	147980.9
77.	56	52	149459.1	101985.4
78.	44	42	99181.62	116581.3
79.	55	53	193375.6	135964.7
80.	48	45	56743.33	94670.76
81.	62	67	80742.42	181206.7
82.	62	56	261744.1	109643.4
83.	N.A.	N.A.	104322.1	90872.9
84.	42	41	210751.4	81562.58
85.	60	64	150169.6	195611.9
86.	52	61	217336.1	228205.7
87.	51	52	188397.4	271943.6
88.	47	44	230692.6	85691.97
89.	55	62	222046.5	137071.1
90.	54	59	148576.2	135809.3
91.	79	84	N.A.	N.A.
92.	51	51	266768	155613.9
93.	48	48	179867.2	265431.5
94.	N.A.	N.A.	82138.9	105756.7
95.	46	50	102047.4	283382.9
96.	70	71	N.A.	N.A.
97.	40	42	40277.36	55567.1
98.	40	42	85601.66	107238.1
99.	N.A.	N.A.	N.A.	N.A.

HMPAO: hexamethylpropylene amine oxime; SPECT: single-photon emission computed tomography; CT: computed tomography; N.A.: not applicable.
